# Mobility-driven estimate reveals elevated air pollution exposure and socioeconomic disparities beyond residence-based approaches in Boston

**DOI:** 10.1038/s41370-025-00820-z

**Published:** 2025-11-08

**Authors:** Nail F. Bashan, Yang Zhang, Michelle L. Bell, Qi R. Wang

**Affiliations:** 1 Department of Civil and Environmental Engineering, Northeastern University, Boston, 02115 MA USA; 2 School of Environment, Yale University, New Haven, 06520 CT USA

**Keywords:** Human mobility–informed PM_2.5_ exposure, Exposure assessment bias, Sociodemographic disparities

## Abstract

**Background:**

Residence-based air pollution exposure assessments ignore daily human mobility and may misrepresent exposure levels and disparities across population groups.

**Objective:**

We hypothesize that incorporating high-resolution mobility trajectories into exposure modeling will reveal higher average PM_2.5_ exposures and uncover sociodemographic disparities that traditional residence-based methods underestimate or conceal.

**Methods:**

We analyzed 155,000 trip records from 990 Boston-area participants (June–December 2023) collected via smartphone GPS, linked to PM_2.5_ measurements from 294 calibrated PurpleAir air quality sensors collected at 2-min intervals. For each stay location, we computed a daily adjusted exposure as the average PM_2.5_ within a 4 km buffer minus the region’s daily average. We compared these mobility-informed exposures to home-based estimates, assessed temporal (weekday vs. weekend, peak vs. off-peak) and spatial variability (Moran’s I), and used weighted least squares regressions and t-tests to evaluate differences across race, income, education, age, and occupation.

**Results:**

Mobility-informed exposures averaged 0.10 µg/m^3^ higher than residence-based estimates on weekdays (up to 0.45 µg/m^3^ on high-pollution days). Employed and higher-income individuals, as well as White participants, experienced significantly elevated exposures during peak travel hours (up to +0.30 µg/m^3^; *p* < 0.01). Spatial clustering of mobility exposures was stronger on weekdays (Moran’s I = 0.4) than weekends (I = 0.2), and regression coefficients confirmed systematic underestimation by traditional methods.

**Significance:**

These findings demonstrate that neglecting mobility systematically underestimates exposure levels and obscures environmental injustices.

**Impact statement:**

Integrating dynamic mobility data with hyperlocal air quality monitoring provides a refined framework for accurate exposure assessment, informing equitable public health policies and targeted interventions.

## Introduction

Air pollution is a major global health challenge, contributing to a wide range of adverse health outcomes [1–4]. In 2021 alone, it was responsible for 8.1 million deaths worldwide, making it the second leading risk factor for mortality, surpassing both tobacco use and dietary risks [5]. An extensive body of research has linked air pollution to a variety of serious health effects, including cardiovascular and respiratory diseases, neurological disorders, and premature death (see [6–8] for comprehensive reviews). Even short-term exposure can trigger severe health issues, particularly among vulnerable populations like children and older populations [9–11]. Given its severe health consequences, accurately estimating and understanding air pollution exposure are crucial for informing public health policies and designing effective mitigation strategies.

The epidemiological analysis of air pollution exposure and its associated health impacts has traditionally relied on temporally aggregated pollution data across certain fixed spatial scales [12–14]. While this approach offers insights into average exposure levels, it may overlook short-term fluctuations and localized variations that can be critical for accurately assessing health risks. Recent hyperlocal studies have demonstrated that air pollution levels can vary significantly within as little as 30 meters [15] and over minute-level timeframes [16]. These variations are especially pronounced in densely populated urban areas, where pollution levels are shaped by the complex physical environment and the multiple, localized sources [17, 18]. This spatiotemporal variability could lead to substantial differences in both short-term and long-term exposures, and unequal levels among different population subgroups, particularly among sociodemographic subgroups with differing vulnerabilities to pollution-related health effects [13]. Evidence shows that racially marginalized populations and individuals with low socio-economic status often bear a disproportionate burden of ambient air pollution, reinforcing patterns of environmental injustice that contribute to persistent health disparities [19–21].

**Fig. 1:**
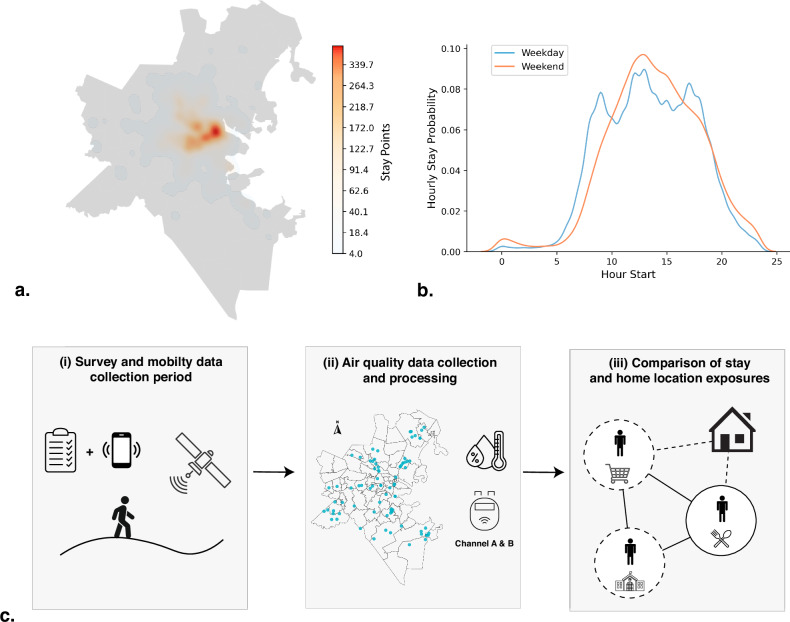
Study Design. **a** Heatmap showing the spatial distribution of stay locations across the Boston Metropolitan Area, with a notable concentration in the downtown region. **b** Temporal Kernel Density Estimation (KDE) distribution of the start hour of stay locations across the city, comparing weekend and weekday patterns. **c** Overview of the methodology: (i) smartphone-based GPS tracking, (ii) air quality data processing using PurpleAir monitors, and (iii) neighborhood-level comparison of stay and home location exposures.

The dynamics of the urban population add another layer of complexity to exposure modeling, with human mobility being a key contributing factor. People spend almost half of their time outside residential addresses [22], and the high spatial variability in daily movements can expose them to high-emission areas (such as industrial zones) making mobility a critical contributor to air pollution exposure [23]. Also, human mobility patterns are highly heterogeneous and unequal between gender groups, urban-rural areas, and countries [24]. In urban settings, city designs play a central role in shaping these patterns, influencing exposure through factors such as the availability and affordability of public transit, proximity to schools and workplaces, parking and traffic policies (e.g., congestion fees), and the safety and accessibility of pedestrian infrastructure. For example, many cities, such as Boston, USA, follow the downtown-centric designs, where people travel longer distances to meet their daily needs and commute to work [25]. This pattern concentrates residential and workplace activity in Boston’s downtown core (Fig. 1a), with visitor activity concentrated during distinct peak hours (Fig. 1b). As a result, air quality tends to deteriorate during morning and afternoon rush hours, when a large portion of the population is on the move [26]. These mobility-driven variations, combined with the spatial heterogeneity of air pollution, can result in exposure misclassification—a challenge that particularly impacts studies pollution-related disparities across different population groups [27].

New technologies have made it possible to address these challenges, but current solutions remain limited in scope and maturity. In particular, GPS tracking technologies and low-cost air quality monitoring systems have allowed for more granular and individual-level exposure assessments by incorporating daily mobility patterns [28, 29]. However, while geo-tagged GPS data are commonly used to understand and predict mobility patterns, their anonymized format presents certain limitations [30, 31]. For instance, inferring sociodemographic characteristics based on the census block group or tract of an individual’s home can lead to biases, especially in densely populated and diverse urban centers [32]. In addition, previous studies combining mobility data with stationary air quality monitors were constrained by limited participant numbers and shorter temporal coverage [33]. Such sparse datasets and low-sample sizes may fail to capture the full temporal variability in daily exposure. Furthermore, GPS traces typically lack behavioral context, such as trip purpose and the environmental characteristics of the stay locations, limiting their utility in fully understanding complex exposure risks [34]. These limitations are further magnified in the post-COVID era, which has seen a fundamental transformation in mobility behavior. The rise of remote work and flexible schedules is the “new norm” [34], which has dramatically altered urban travel behaviors, requiring updated and comprehensive travel surveys to reflect current exposure dynamics beyond what was captured in pre-pandemic research [33, 35]. Together, these challenges underscore the importance of collecting and analyzing detailed mobility data with comprehensive, individual-level sociodemographic information. Integrating such details allows researchers to study different population subgroups more accurately and address disparities that cannot be captured by anonymized datasets alone.

In this study, we hypothesize that neglecting human mobility underestimates the air pollution exposure and associated disparities. To test our hypothesis, we use a unique data set that captures high-resolution mobility trajectories of urban dwellers, enriched with detailed individual-level sociodemographic information. The data, collected through the BostonWalks study over a seven-month period in 2023 [36], include 155,000 trip records from 990 residents within the Boston metropolitan area, spanning from June 1 to December 31, 2023. We match these mobility data with air quality measurements obtained from third-party PurpleAir sensor networks within the same region to analyze and quantify air pollution exposure and its heterogeneity across individuals (Fig. 1c). Our findings shed light on how and to what extent traditional residence-based exposure assessments underestimate actual air pollution levels across Boston. Moreover, we demonstrate that these biases vary significantly by sociodemographic groups, highlighting the critical importance of accounting for dynamic mobility patterns when informing public health and environmental justice initiatives.

## Methods

### Study area and data sources

We concentrated our analysis on the inner core of the metropolitan area of Boston, Massachusetts, USA defined by the Metropolitan Area Planning Council (MAPC) and commonly referred to as the Inner Core Committee (ICC) subregion. This zone encompasses 21 municipalities and has a population of roughly 1.8 million living in approximately 720,000 households. For our analysis we combined two main datasets: smartphone-based human mobility data from the BostonWalks study and PM_2.5_ measurements from PurpleAir air quality sensors collected at 2-min intervals. These data were collected during the same time window (June 1–December 31, 2023) to facilitate integrated exposure assessment.

BostonWalks is a GPS-tracking study that collected detailed travel data for 990 participants who were recruited from the study area. Participants were recruited using quotas based on the 2022 American Community Survey (ACS) to ensure balanced representation across age, gender, income, race, ethnicity, and education. Final weights were then applied to correct any remaining under-representation. Participants downloaded a dedicated smartphone application (Catch-My-Day app, available for iOS and Android) that automatically recorded their trips, modes of transportation (car, public transit, walking, biking, etc.), and stay locations. The Catch-My-Day app applies its backend algorithm to incoming smartphone sensor data, conducting an initial round of outlier filtering and trip segmentation for accurate location data. In addition, user surveys captured sociodemographic indicators including race, income, education, age, and occupation. These indicators were linked to the GPS data using unique participant IDs, enabling both individual and subgroup-level analyses.

The final BostonWalks dataset yielded approximately 155,000 individual trip records and corresponding stays. The study required each participant to record their mobility for at least 14 consecutive days during the study period. Trips are defined as continuous movements between origin and destination points, as detected by the backend algorithm of the Catch-My-Day platform used in the BostonWalks study. In our analysis, we removed participants with fewer than 10 recorded tracking days to minimize the impact of sparse travel data. We retained only stay events lasting between 30 min and 5 h, assuming these represent meaningful exposures beyond brief stops (SI Fig. S11). Implementing a minimum duration threshold for stay locations follows established methodological practices in mobility research, preventing the misclassification of brief pauses (such as traffic light stops or brief drop-offs) as significant exposure locations [31, 37]. This approach enhances analytical precision by avoiding the overweighting of negligible exposure periods and focuses the analysis on locations where meaningful exposure accumulation occurs. To focus on periods when individuals are likely to be outdoors, we limited our analysis to stay locations recorded between 6:00 a.m. and 10:00 p.m.

PurpleAir is a crowdsourced network of low-cost air quality monitors. It is equipped with two laser particle counters (PMS-5003) that provide PM_2.5_ readings in two channels (A & B), alongside separate sensors that measure pressure, temperature, and humidity. We used PM_2.5_ data collected at 2-min intervals from all publicly accessible PurpleAir sensors within our study area during the same time period. Readings from 294 sensors were corrected and cleaned according to the calibration factors to account for known biases [38]. We removed temperature outliers (values below −200°C or above 1000°C) and dropped observations where both channels failed to record at a given timestamp. Each raw PM_2.5_ reading was then calibrated (combining sensor channels A and B) and adjusted for humidity. Observations were discarded if the difference between channels A and B exceeded 5 µg/m^3^ or 61% of the measurement value, indicating a channel disagreement that compromised data accuracy. Finally, we excluded sensors that recorded fewer than 75% of the observations of the highest-recording sensor during the study period, as these were deemed to have insufficient coverage. This resulted in a clean, uniformly processed dataset for further analysis. To assess the quality of the low-cost sensors, we compared daily PM_2.5_ averages from the PurpleAir monitors with reference-grade monitors (FRM/FEM) in the study area. The results showed strong agreement, supporting the use of PurpleAir data for exposure estimation (SI Fig. S10). The resulting dataset covers most ZIP codes in the study region and reflects both short-term fluctuations and spatial heterogeneity in PM_2.5_ concentrations.

### Exposure assessment

We calculated the daily adjusted stay location exposure to quantify short-term exposure to ambient PM_2.5_ in any place where a participant remains for at least 30 min. Since our study spanned 7 months and each participant was tracked with a median tracking period around 23 days, relying on raw PM_2.5_ readings could introduce bias due to day-to-day variability. For instance, if a high-pollution week coincides with a larger sample from a particular group, that group might be inaccurately classified as highly exposed. To address this, we defined a mean-adjusted exposure metric that normalizes readings across time, ensuring a consistent estimate of daily exposure differences across groups. We calculated a daily mean PM_2.5_ value each day using all sensors in the region and subtracted this mean from the measured stay-level PM_2.5_ to derive a relative exposure metric showing a stay location’s pollution compared to a given day’s overall baseline. This adjustment approach aligns with established methodologies in the literature. It has been used to define PM_2.5_ anomalies as deviations from location-specific median values calculated over recent months during non-smoke days, enabling more robust comparisons across temporal and spatial contexts [39].

We implemented a spatial buffering approach that directly integrates empirical measurements from fixed-site outdoor air quality monitors. Because our dataset does not distinguish between indoor and outdoor stay locations, we used outdoor air quality measurements as a proxy for all stays. This approach is supported by prior research indicating that indoor air quality is generally highly correlated with outdoor levels [40, 41]. We followed a neighborhood-based representativeness scale [42] by placing a 4 km circular buffer around each GPS-derived stay location. This spatial scale refers to concentrations within some extended area of the city that has relatively uniform land use and similar surface characteristics. For each stay location, we identified nearby monitors located within a 4 km Euclidean distance. If more than one monitor was found within this buffer, the average PM_2.5_ concentration across all such monitors was used to represent exposure at that location (SI Fig. S1a):

$${Exposure}({s}_{k},t)=\,\frac{1}{n} {\sum}_{i=1}^{n}{{PM}}_{2.5}({m}_{i},t)$$

where the exposure at stay point *s*_*k*_ at time *t* is calculated as the average PM2.5 concentration from all n monitors located within 4 km of *s*_*k*_, using their values at time *t*.

This method allows us to assign temporally matched, location-specific exposure estimates based solely on observed monitor data, without relying on interpolated surfaces or land-use regression (LUR) models. This restricts our input data to observed monitor values, providing a transparent and conservative exposure estimation framework.

While prior studies have commonly used modeled pollution fields or LUR-based estimates incorporating land-use characteristics within buffers [43], our approach directly links raw monitor data to individual mobility patterns using spatial proximity alone. Methodologically, our approach aligns more closely with studies such as [44] which derived daily exposures by averaging modeled pollution values within GPS-based buffers. As a result, 92% of the 63,361 identified stay locations were spatiotemporally matched with at least one PurpleAir monitor, and nearly 76% were paired with four or more monitors. This multi-monitor matching approach improves accuracy by reducing reliance on a single sensor (SI Fig. S1b).

Finally, to represent the exposure difference between individual home locations and stay points, we compared the average PM_2.5_ readings within a 4 km radius of each stay location to those around an individual’s home over the same period. We identified individuals’ home locations using raw GPS data by filtering each user’s trajectory for visits during a defined nighttime interval (10:00 p.m.–6:00 a.m.) and selecting the latitude–longitude pair most frequently visited during that period [45]. The difference between stay exposure and home exposure indicates whether traveling away from home leads to higher or lower pollution levels for each participant.

### Statistical analysis

Our initial exploratory analysis involved computing summary statistics and generating visualizations to explain the temporal and spatial distributions of exposure levels. Time-series plots and geographic maps were produced to identify temporal trends and spatial disparities in pollution exposure. To assess differences in exposure across various travel behaviors, we conducted t-tests comparing mean values (µ₁ ≠ µ₂) between different exposure aggregations, which confirmed the heterogeneity in pollution exposure associated with distinct trip purposes during weekdays or weekends. We used Moran’s I statistic to evaluate spatial autocorrelation, where higher Moran’s I values (with significant p-values) indicate stronger spatial clustering in exposure levels.

We performed Weighted Least Squares (WLS) regressions to examine the relationship between daily adjusted stay location exposure and sociodemographic predictors, including race, income, age, and education. Our weighting scheme corrected for over- or under-representation of specific demographic groups in the final sample. Average exposures were computed separately for weekdays and weekends, and two models were developed accordingly. In each model, the regression parameters represent the relative difference compared to the reference category, with p-values and standard deviations reported. All sociodemographic variables were self-reported by participants.

## Results

In our analysis, we examined two primary metrics. First, we defined a “stay location” as any place where a participant remains for at least 30 min. The daily adjusted stay location exposure was calculated by averaging the PM_2.5_ readings from all PurpleAir sensors within the neighborhood level (4 km radius) of each stay location, then subtracting the daily mean PM_2.5_ (across all sensors) to account for overall daily fluctuations. Second, we evaluated the exposure shift due to mobility by comparing average PM_2.5_ levels around each stay location to those around the participant’s home, both calculated within the same 4 km radius and time window. The resulting difference reflects how exposure levels would change if the individual had stayed home instead of visiting the recorded location.

### Spatiotemporal variation of exposure

**Fig. 2:**
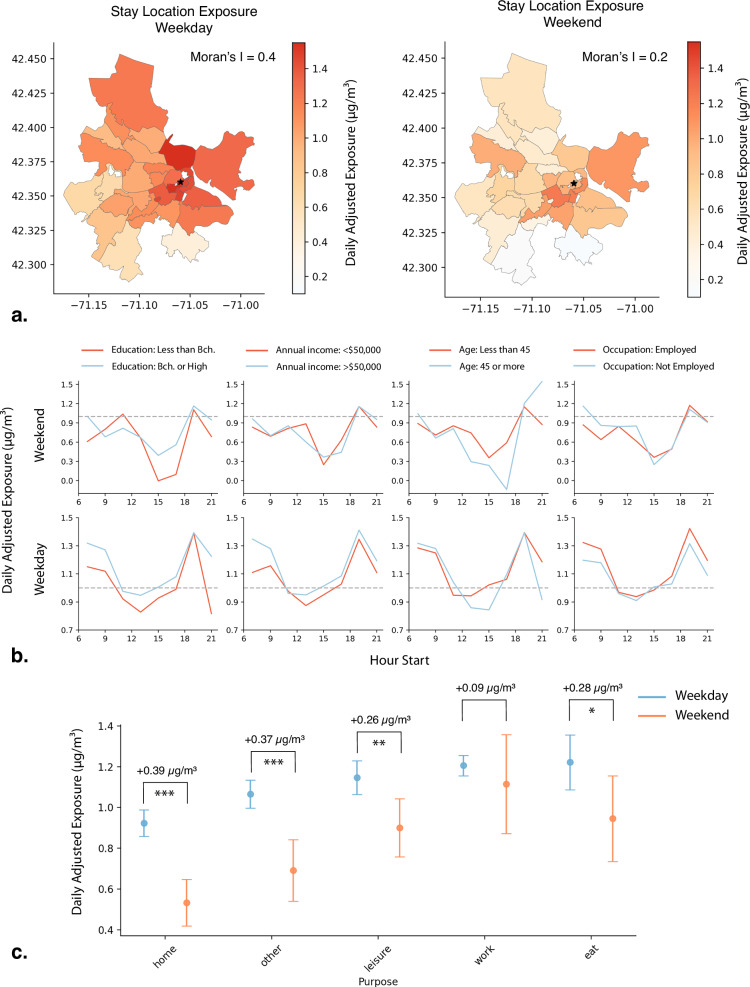
Spatiotemporal Variation of Exposure. **a** Spatial correlation (Moran’s I) among the 30 most visited ZIP codes in the Boston Metropolitan Area (downtown marked with a star). Correlation is higher for stay location exposures during weekdays (left) and lower during weekends (right). **b** Exposure levels across different socioeconomic groups, shown relative to the daily average during weekends (top) and weekdays (bottom). **c** Exposure relative to daily averages across various self-reported trip purposes, with changes in averages (µg/m^3^) compared between weekdays and weekends with 95% confidence intervals. Differences (µ₁ ≠ µ₂) were tested for statistical significance using a *t*-test (*p < 0.05, **p < 0.01, ***p < 0.001), where µ₁ and µ₂ represent the average values for two different groups.

We found that mobility-related exposure during weekdays exhibited a stronger spatial correlation (Moran’s I = 0.4) in the 30 most visited ZIP codes in Boston Metropolitan Area (Fig. 2a, Table S1). In contrast, the spatial correlation during weekends was lower (Moran’s I = 0.2), suggesting a more heterogeneous exposure across neighborhoods during the weekends. Most neighborhoods experience higher mobility-related exposures on weekdays, likely due to a combination of increased pollutant levels and more commuting and work-related travels. In contrast, weekend mobility is more dispersed both spatially and temporally, making exposure patterns harder to predict and contributing to greater heterogeneity. Note that the shift from weekday to weekend exposure was inconsistent, some ZIP codes saw larger reductions while others experienced smaller or negligible changes (SI Fig. S2). This inconsistency reflects non-uniform weekday–weekend mobility patterns across ZIP codes. For example, even among the three most visited ZIP codes adjacent to each other (02139, 02215, and 02116) the weekday exposures were 0.33 µg/m^3^, 0.37 µg/m^3^ and 0.15 µg/m^3^ higher, respectively, than weekend exposures, illustrating how localized patterns influence exposure dynamics.

When comparing exposure differences across temporal scales for different population groups, we observed a similar pattern of increased exposure during the morning and afternoon peak hours, aligning with periods of higher traffic volume and thus traffic-related pollution (Fig. 2b). However, these temporal differences varied more noticeably across different socioeconomic groups. Specifically, during weekdays, individuals with higher education levels and higher incomes were exposed to up to 0.3 µg/m^3^ higher than the daily average air pollution levels during morning peak hours, likely due to commuting patterns that bring them near highways and high-density areas. Similar pattern was observed in the employed population during weekdays with exposure differences up to 0.1 µg/m^3^. Age, however, did not show any temporal differences compared to weekday and weekends. Among different racial groups, we observed that on weekday mornings White individuals experienced daily adjusted exposures that were 0.05 µg/m^3^ higher than those for Black/African-American groups and 0.25 µg/m^3^ higher than those for Asian/Asian-American groups (see Fig. S3).

Finally, we compared weekday and weekend exposure differences for different self-reported trip purposes and evaluated their statistical significance using a *t*-test. Individuals who engaged in activities corresponding to the top three reported trip purposes (other, leisure, and dining activities) exhibited significant weekday versus weekend differences, with weekday exposures exceeding weekend exposures by 0.37 µg/m³ (*p* < 0.001) for “other”, 0.26 µg/m³ (*p* < 0.01) for “leisure”, and 0.28 µg/m³ (*p* < 0.05) for “dining” (Fig. 2c). In contrast, work-related trips did not show significant differences (*p* > 0.05). Note that workplace locations are highly concentrated in specific areas, whereas trips for other purposes are more spatially dispersed (SI Fig. S4), which may explain the limited variability in work-related exposures.

### Sociodemographic variation of exposure

We conducted a weighted least squares regression to examine the relationship between air pollution exposure and sociodemographic characteristics. In Table 1, each coefficient estimates the difference in average exposure compared to a predefined reference group. The reference groups are: White participants (Race), individuals with less than a bachelor’s degree (Education), those earning under $50,000 (Income), individuals aged 45 or younger (Age), employed individuals (Occupation), and those identifying as Hispanic (Ethnicity). Statistically significant results (*p* < 0.05) are marked with an asterisk, indicating that the corresponding variable has a statistically significant association with exposure levels.

**Table 1:** Weighted least squares regression for weekend and weekday exposures relative to daily averages.

	Weekend Exposures	Weekday Exposures
Intercept	0.7069^*^	1.0679^*^
	(0.042)	(0.019)
	*P* = 0.000	*P* = 0.000
Race: Black or African-American	0.4411^*^	-0.1177^*^
	(0.053)	(0.022)
	*P* = 0.000	*P* = 0.000
Race: Asian or Asian-American	0.0477	-0.0394
	(0.057)	(0.022)
	*P* = 0.398	*P* = 0.076
Race: Other	-0.0321	0.0625^*^
	(0.047)	(0.019)
	*P* = 0.496	*P* = 0.001
Education: Bachelor’s degree or more	0.2635^*^	0.0921^*^
	(0.034)	(0.014)
	*P* = 0.000	*P* = 0.000
Annual income: $50,000 or more	-0.0987^*^ (0.033)	0.0578^*^ (0.014)
	*P* = 0.003	*P* = 0.000
Age: 45 or more	-0.1071^*^	-0.0557^*^
	(0.028)	(0.012)
	*P* = 0.000	*P* = 0.000
Occupation: Not Employed	0.0104	-0.0448^*^
	(0.030)	(0.013)
	*P* = 0.732	*P* = 0.001
Ethnicity: Not Hispanic	-0.2328^*^	-0.0405^*^
	(0.036)	(0.016)
	*P* = 0.000	*P* = 0.012

Explanatory features include race, education, income, age, and occupation. (* denotes significant regression coefficients with *p* < 0.05; standard deviations are shown in parentheses).

Our analysis reveals distinct patterns of pollution exposure shaped by race and socioeconomic factors. We highlight a few key findings. First, Black or African-American participants experienced lower exposure on weekdays compared to the White reference group; however, this trend reversed on weekends, suggesting that differences in daily routines or neighborhood environments may contribute to higher weekend exposure (SI Fig. S6). Furthermore, individuals with higher education levels (often associated with employment in urban centers) consistently encountered elevated pollution exposure, likely reflecting increased time spent in densely populated or traffic-heavy areas. In contrast, non-employed participants showed significantly lower weekday exposures, potentially due to reduced commuting and fewer work-related trips. These patterns can be attributed to the distinct spatiotemporal mobility behaviors observed among these groups (SI Fig. S5). For example, higher-educated and employed individuals tend to have increased activity in downtown areas during morning peak hours and lunchtime. Additionally, while the overall employment rate was 72% in the population, it rose to 80% for downtown travelers, underscoring how the timing and location of travel influence exposure levels.

It is worth noting that there are high interdependencies among socioeconomic factors (SI Fig. S7). For instance, 90.5% of individuals with education beyond a bachelor’s degree were employed, 87% of the White population was highly educated, and 77% reported an annual income exceeding $50,000. These overlapping characteristics highlight the need to consider such correlations when interpreting our results.

### Mobility variation of exposure

**Fig. 3:**
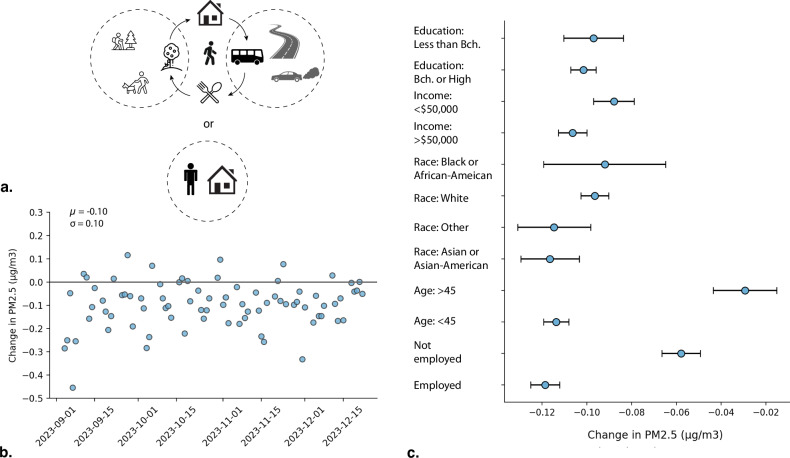
Mobility Variation of Exposure. **a** Our methodology analyzes the exposure difference between each user’s stay and home locations during the same time intervals. **b** Daily exposure difference across the entire population based on residence compared to exposure incorporating mobility patterns. **c** Personal average exposure differences across various demographic groups if estimates were based on peoples’ home location, with 95% confidence intervals.

To assess the bias of using home-based estimates for air pollution exposure—a common approach in exposure assessment studies [46, 47]—we compared the PM_2.5_ levels at each recorded stay point to the pollution level at the individual’s home location. Our analysis shows that relying solely on home location underestimates actual exposure: on average, weekday exposure would be about 0.1 µg/m^3^ lower if based only on home locations. On certain weekdays, this difference reached up 0.45 µg/m^3^. The difference rarely turns positive, indicating that people are generally exposed to higher pollution levels when they travel away from home (Fig. 3b). This highlights that actual exposure is typically higher than estimates based on residential location alone, and that this discrepancy varies day by day. We also find a moderate negative correlation (*r* = -0.57) between daily PM_2.5_ averages and the exposure difference (SI Fig. S8), suggesting that the underestimation is more pronounced on days with higher pollution.

However, this change in exposure varies across socioeconomic group (Fig. 3c). For middle-aged and older adults (age >45), minimal difference was observed in pollution exposure calculated incorporating mobility versus calculated based on residence. On average, individuals over 45 years traveled 10.1 km away from home, compared to 6 km for those under 45 (SI Fig. S9). This indicates that while younger individuals travel shorter distances, the pollution levels at their destinations vary more widely. In contrast, although middle-aged and older adults traveled farther, the spatiotemporal pollution levels at their destinations were more homogeneous, resulting in smaller differences between home-based and actual exposure estimates. A similar pattern emerged for unemployed individuals. In contrast, employed people showed the largest difference, with residence-based estimates averaging 0.12 µg/m³ lower than mobility-based exposure.

## Discussions

Several limitations of this study should be acknowledged. First, our reliance on outdoor air quality measurements as proxies for total exposure does not capture the full variability of indoor air quality, which can differ significantly by setting and activity. Second, our mobility dataset spans only June through December 2023, potentially missing important seasonal variations in both mobility patterns and pollution levels that occur during winter and spring. To advance this effort, it is essential to design travel surveys that strategically capture periods of fluctuating pollution levels and shifting travel behavior, while also identifying whether participants are indoors or outdoors. Combining this level of spatiotemporal and contextual accuracy with building-specific indoor/outdoor pollution infiltration ratios can yield a more precise understanding of individual exposure differences.

While we adopt established approaches for data cleaning and calibration, low-cost sensors, such as PurpleAir sensors, may still introduce systematic biases compared to regulatory-grade monitors. It is important to better link the measurement accuracy of low-cost monitoring systems with the economic and health impacts of changing pollution levels in communities. Also, while our use of a 4 km buffer enhances neighborhood representativeness, this form of spatial aggregation can smooth over hyperlocal pollution gradients that may substantially influence individual exposure. Future research should expand upon this work by exploring these patterns across different cities with varying urban design conditions.

Despite these limitations, our study represents a pioneering effort to quantify air pollution exposure biases across diverse spatiotemporal conditions and demographic groups in the Boston area. To do this, we integrated high-resolution human mobility data from smartphone-based travel surveys with calibrated PM_2.5_ measurements from a network of low-cost air quality sensors. Our findings provide empirical evidence that conventional residence-based exposure assessment methods systematically underestimate actual exposures that account for daily mobility by an average of 0.1 µg/m^3^, with differences reaching up to 0.45 µg/m^3^ on high-pollution days. These disparities also vary across different sociodemographic groups, with differences of up to 0.12 µg/m^3^. Employed individuals experience the largest discrepancies between mobility-based and residence-based exposure estimates.

Another key finding of this study is that the temporal and spatial heterogeneity of exposure reveals that air pollution disparities are shaped not only by where people live, but also by where they go and when they make these trips. Spatial variations in exposure can be up to 1.0 µg/m^3^ across different ZIP codes in the metropolitan area and temporal variations up to 0.3 µg/m^3^ among different sociodemographic groups driven by differences in daily visitation patterns. The variations highlight complex relationships between individual characteristics and pollution burden. Specifically, our results show that employed individuals and those with higher education levels experience distinct exposure patterns that would not be accurately captured by residence-only assessments or anonymized mobility data without sociodemographic information.

Although this work focuses on Boston as a city testbed, our methodologies and findings can be generalized to many cities worldwide where human mobility plays an important role but has been historically overlooked in air pollution exposure estimates. Our findings have important policy implications for urban planning, public health assessments, and environmental justice initiatives. Targeted interventions during peak exposure periods, improved public transportation options in highly polluted corridors, and more flexible work schedules help mitigate exposure disparities. The economic value of 1 µg/m^3^ reduction in PM_2.5_ is substantial, with an estimated average benefit of $1.6 billion in U.S. cities alone [48]. In China, previous research estimates that 21,000–36,000 deaths per year could be prevented by reducing PM_2.5_ exposure by 1 µg/m^3^ [49]. Additionally, by linking our refined, mobility-based exposure estimates with health outcome data, researchers and policymakers can more accurately quantify the public health and economic impacts of air pollution exposure. This approach will thus enable more effective and equitable interventions, particularly in high-exposure urban zones.

## Supplementary information

Supplementary Information

## Data Availability

Sociodemographic and identifiable information used in this study cannot be shared publicly according to our Institutional Review Board (IRB) agreement. However, the data are open for researchers and can be requested following this website: https://web.mit.edu/bostonwalks/. We included the corresponding code so that users can run everything immediately upon downloading the GitHub repository. Air quality data we used are obtained from PurpleAir API.

## Code availability

Codes to reproduce all results in the paper are available at: https://github.com/nnbashan/exposure_bias/.
