# Supplementary material for: Mobility-driven estimate reveals elevated air pollution exposure and socioeconomic disparities beyond residence-based approaches in Boston
Source: J Expo Sci Environ Epidemiol. 2025 Nov 8;36(2):267–75. doi: 10.1038/s41370-025-00820-z (PMC12960195; doi:10.1038/s41370-025-00820-z)
Supplement: Supplementary file 1 — Supplementary Information [file 41370_2025_820_MOESM1_ESM.docx]

**Supplementary Information**

Mobility-driven estimate reveals elevated air pollution exposure and socioeconomic disparities beyond residence-based approaches in Boston

Nail F. Bashan^1^, Yang Zhang^1^, Michelle L. Bell^2^, Qi R. Wang^1*^

^1^ Department of Civil and Environmental Engineering, Northeastern University, Boston, 02115, MA, USA

^2^ School of Environment, Yale University, New Haven, 06520, CT, USA

Corresponding author(s). E-mail(s): [q.wang@northeastern.edu](mailto:q.wang@northeastern.edu)

Contributing authors: [bashan.n@northeastern.edu](mailto:bashan.n@northeastern.edu); [ya.zhang@northeastern.edu](mailto:ya.zhang@northeastern.edu); [michelle.bell@yale.edu](mailto:michelle.bell@yale.edu)

Keywords: Human mobility–informed PM_2.5_ exposure; Exposure assessment bias; Sociodemographic disparities


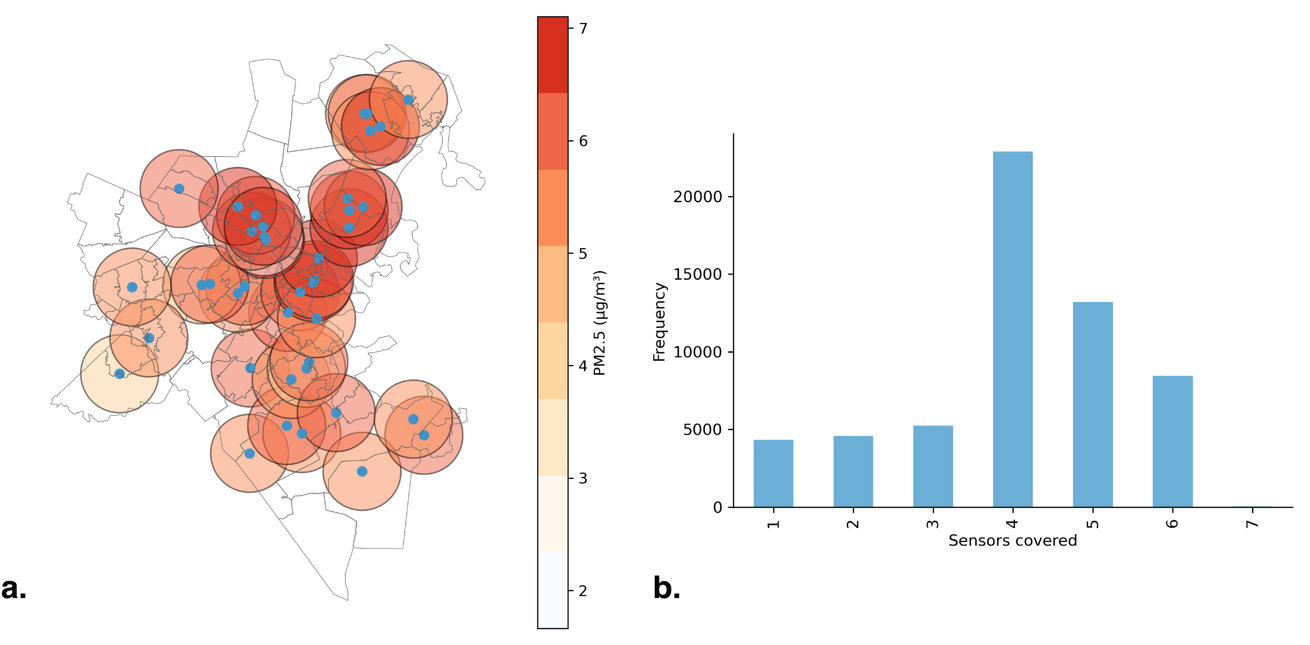


**Figure S1. Spatial distribution of PurpleAir monitors and intersection with stay points.
(a),** Map of the 55 PurpleAir sensors remaining after data cleaning, each surrounded by a 4 km buffer. The buffer regions are shaded according to the average PM_2.5_ levels measured from June to December 2023. **(b),** Histogram showing the number of stay points based on how many sensor buffers they intersect. Incorporating data from multiple sensors enhances exposure estimates, given the uncertainties associated with relying on a single monitor.


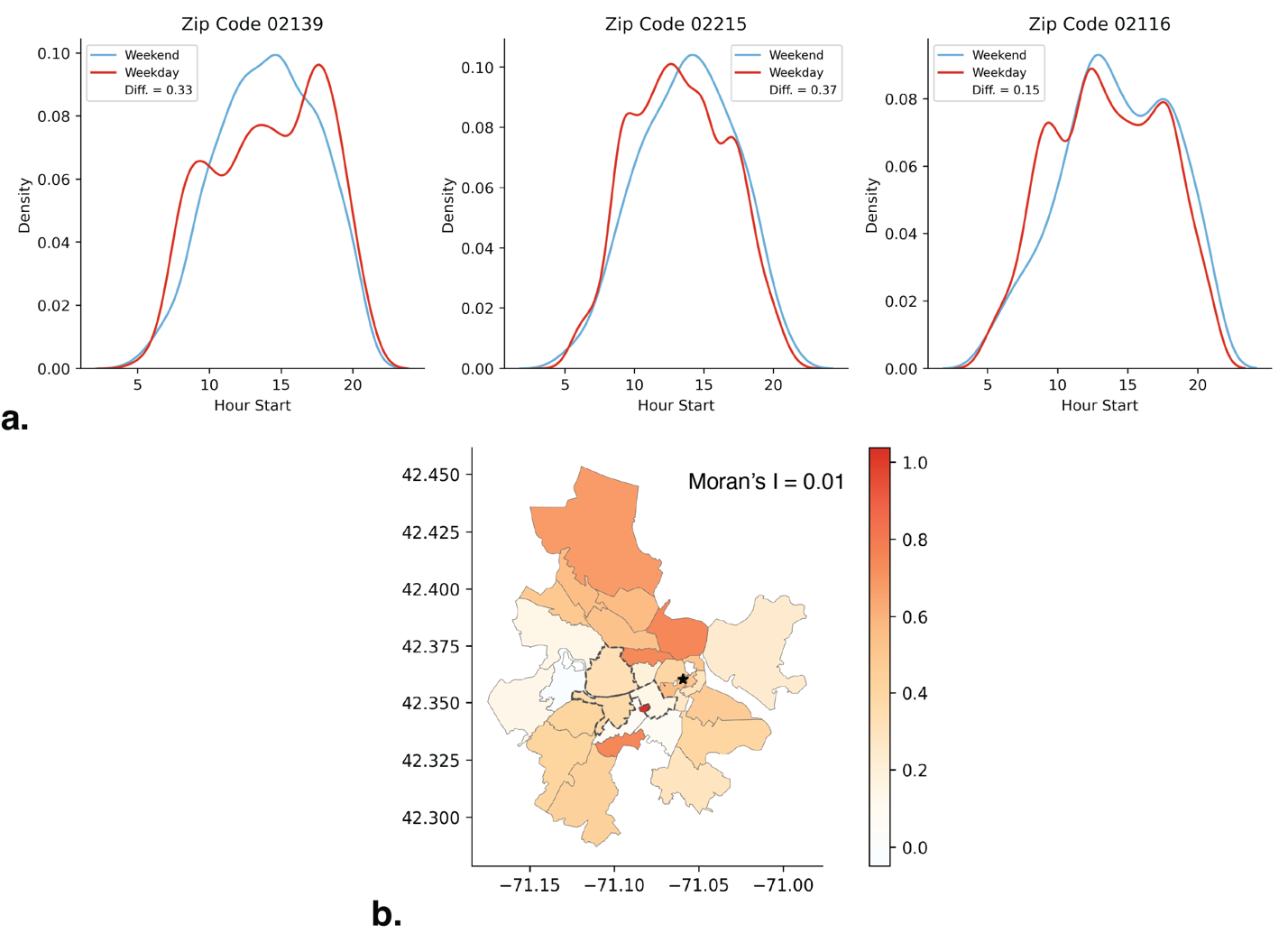


**Figure S2. (a), Temporal stay location probabilities for the top three most frequently visited ZIP codes in the Boston Metropolitan Area.** Each subplot compares weekend and weekday activity patterns using Kernel Density Estimation (KDE) curves. The legend box displays the difference in pollution exposure (diff.) within the same ZIP code across weekends and weekdays, illustrating the variability in spatiotemporal visitation patterns. **(b), The exposure difference between weekdays and weekends** among the top 30 most visited ZIP codes in the Boston Metropolitan Area (downtown marked with a star). The differences do not show a spatial correlation with Moran’s I = 0.01. Three most visited ZIP-codes (02139, 02215, and 02116) are highlighted with dashed lines


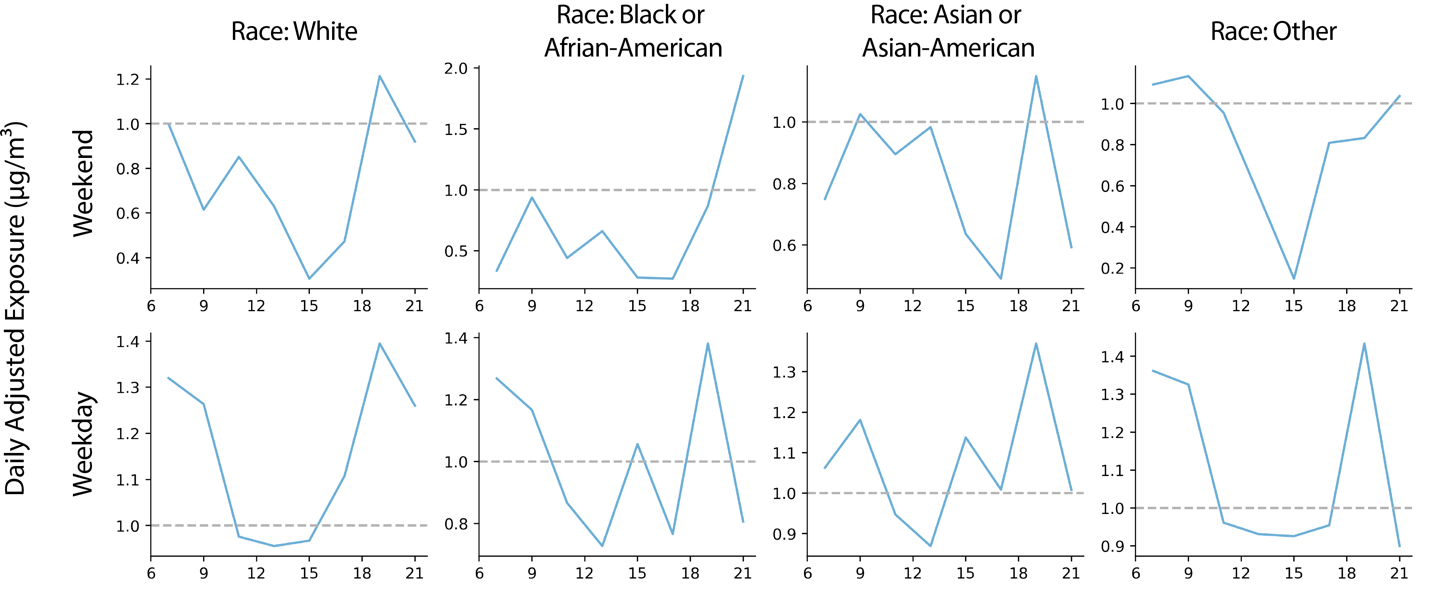


Figure S3. Exposure levels across different race groups, shown as relative to the daily average during weekends (top) and weekdays (bottom).


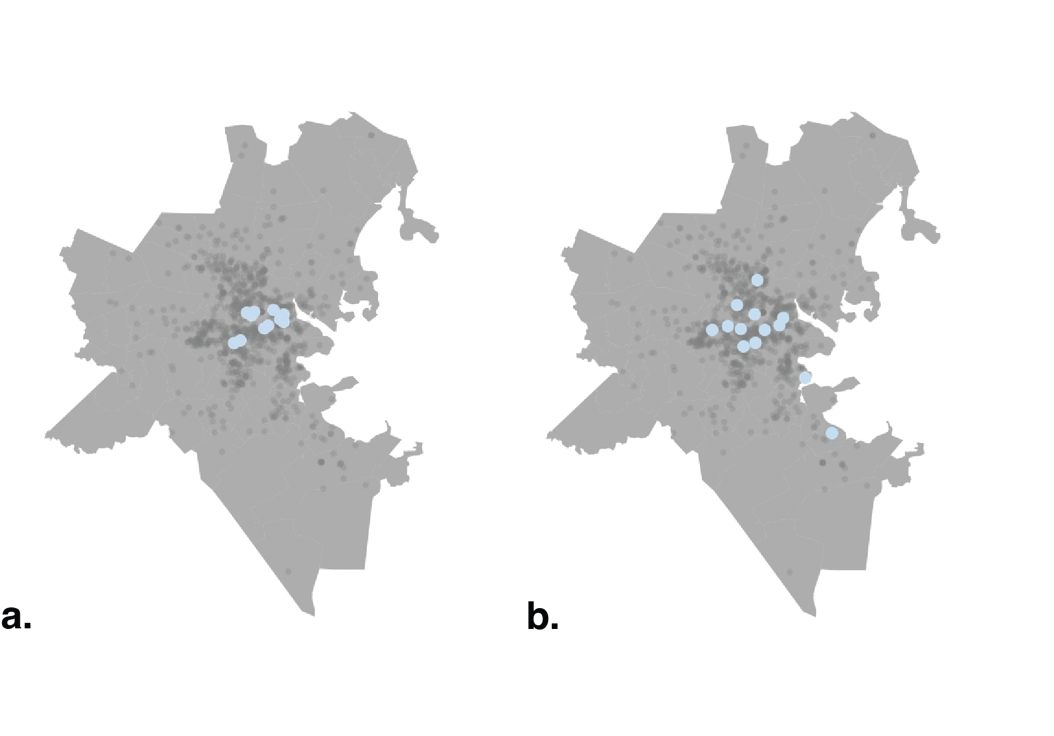


**Figure S4. DBSCAN clustering with mobility data. (a), Work locations (blue) are highly concentrated in the downtown area (**an area characterized by higher population density and elevated pollution levels)**. (b), Other stay locations (blue) exhibit a broader spatial distribution compared to work locations, while home locations (gray) are dispersed throughout the Boston Metropolitan Area.**


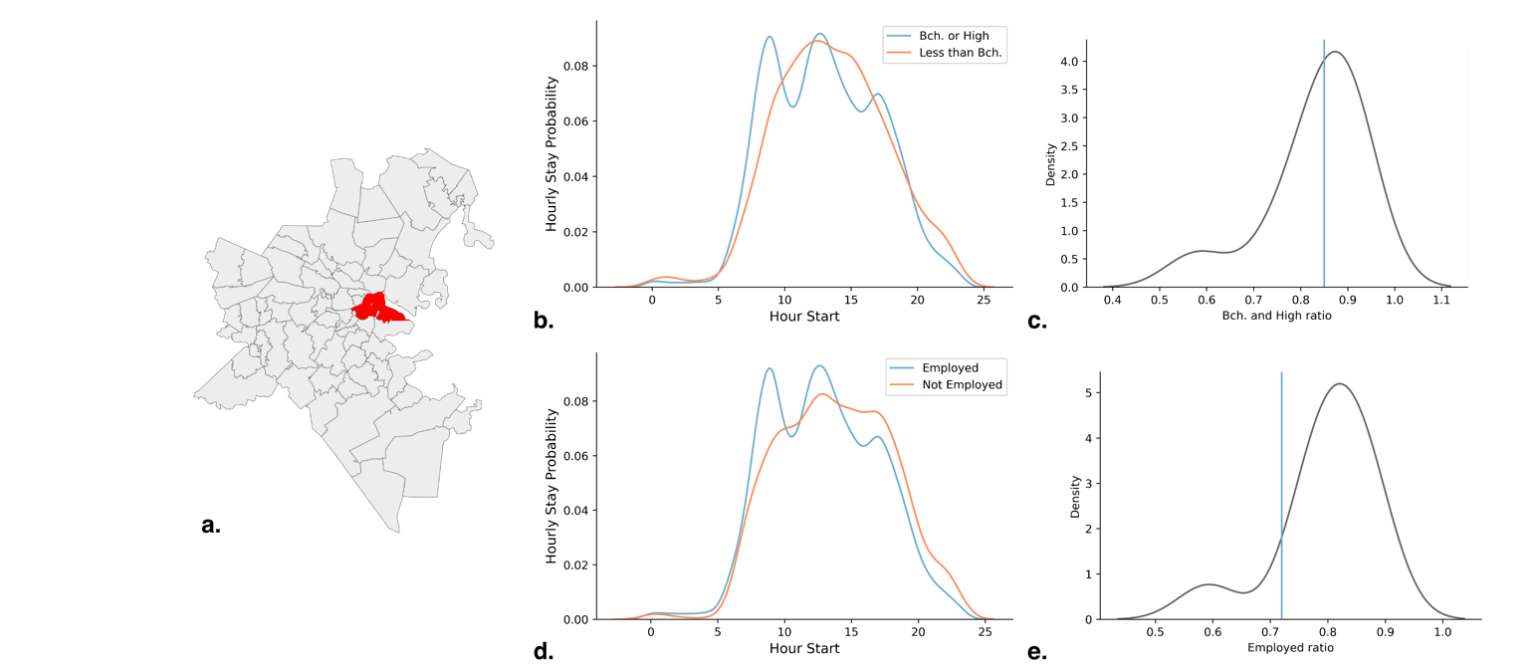


**Figure S5. Mobility Patterns in Downtown Boston** **(a),** Boston Metropolitan Area with downtown ZIP codes highlighted in red. **(b)** and **(d),** Hourly stay probabilities in the downtown area, compared across education levels (**b**) and employment status (**d**).
**(c)** and **(e),** Distributions of visitors’ education (**c**) and employment ratios (**e**) within the downtown area, contrasted with the overall population average (blue line).


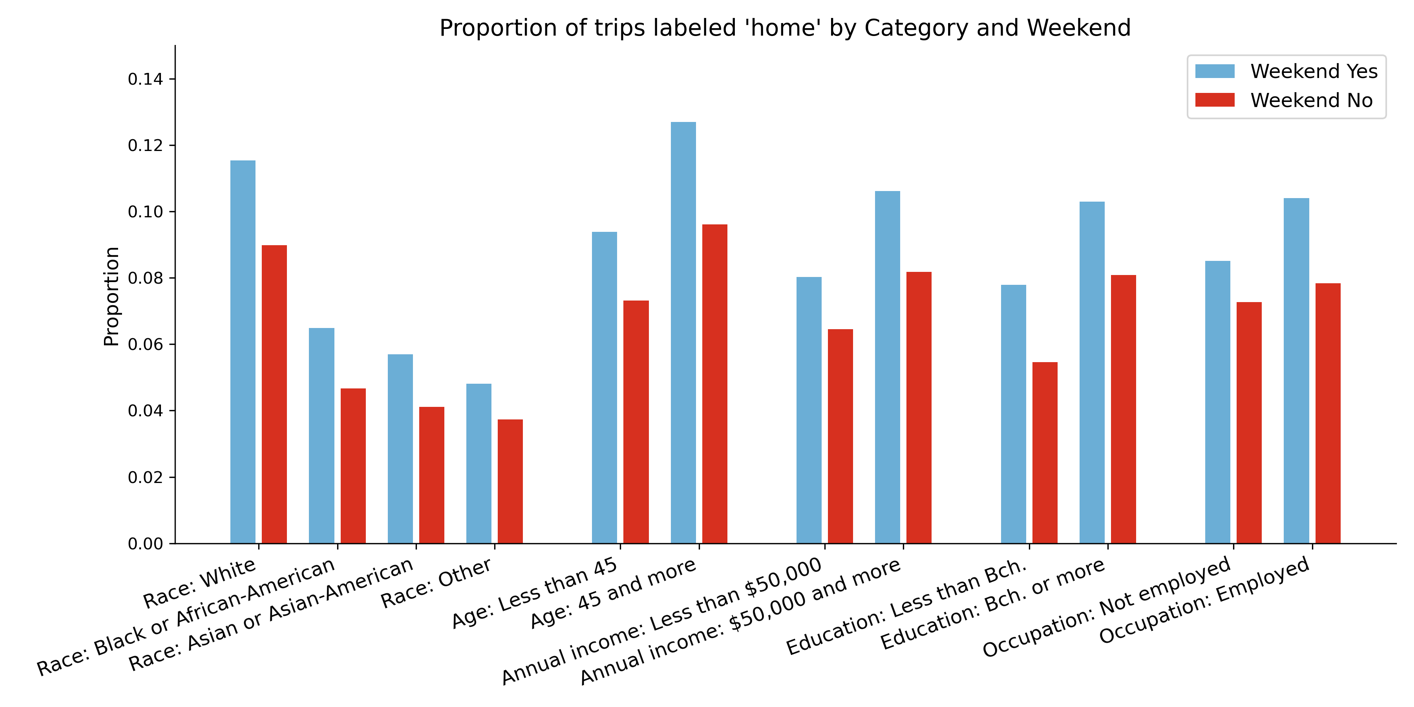


**Figure S6. Proportion of trips labeled “home” by sociodemographic categories across weekdays** (red) **and weekends** (blue)**.** The observed differences highlight how sociodemographic attributes can influence individual mobility patterns and their likelihood of remaining at home on weekdays versus weekends. We can see that while white population had usually higher home labeled stay points both in weekday and weekends, this ratio was lower for other race groups.


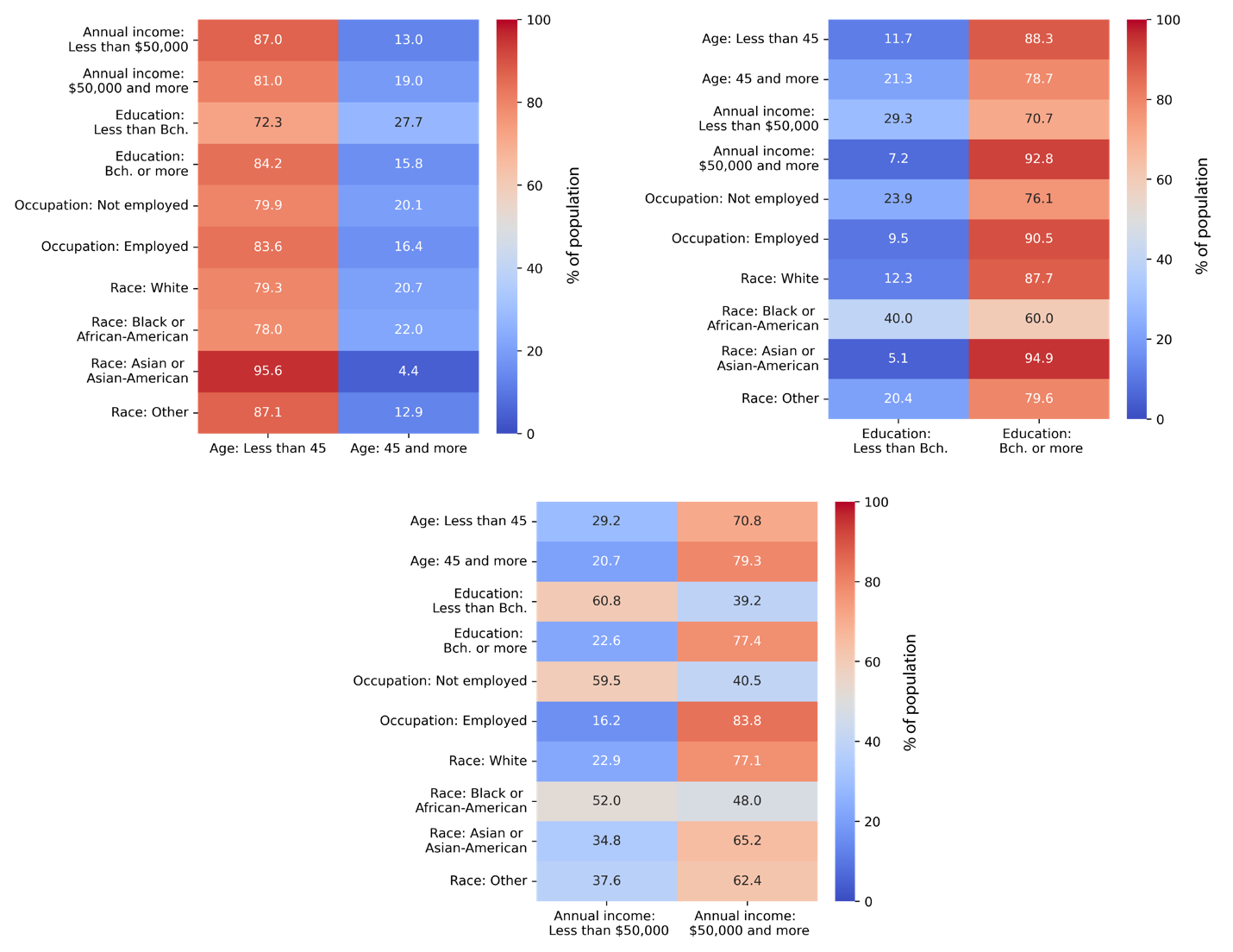


**Figure S7. Correlation heatmap of sociodemographic features.** Each cell represents the unweighted proportion of a y‐variable within an x‐category, offering a visual summary of the overlaps among various demographic attributes.


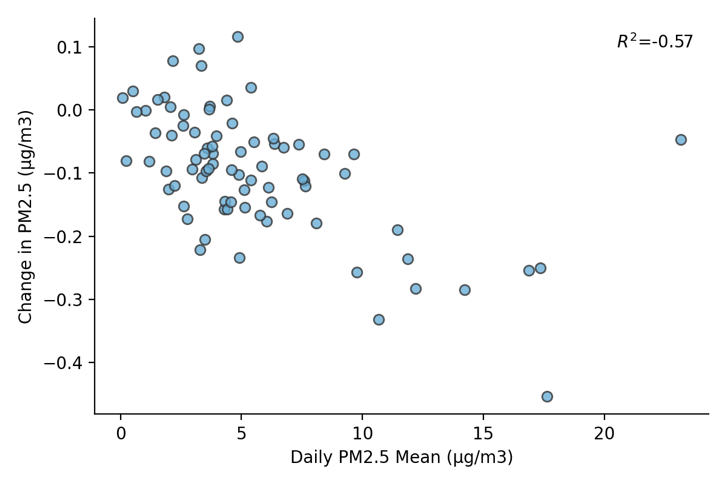


**Figure S8. Relationship between daily mean PM_2.5_ levels and the difference in exposure estimates (home-based vs. mobility-based).** Each point represents a single day, with the y-axis indicating how much daily exposure estimates shift when considering individuals’ actual travel routes rather than assuming they remain at home. The r = -0.57 highlights a moderate correlation, suggesting that higher pollution days yield larger discrepancies between the two exposure estimation methods.


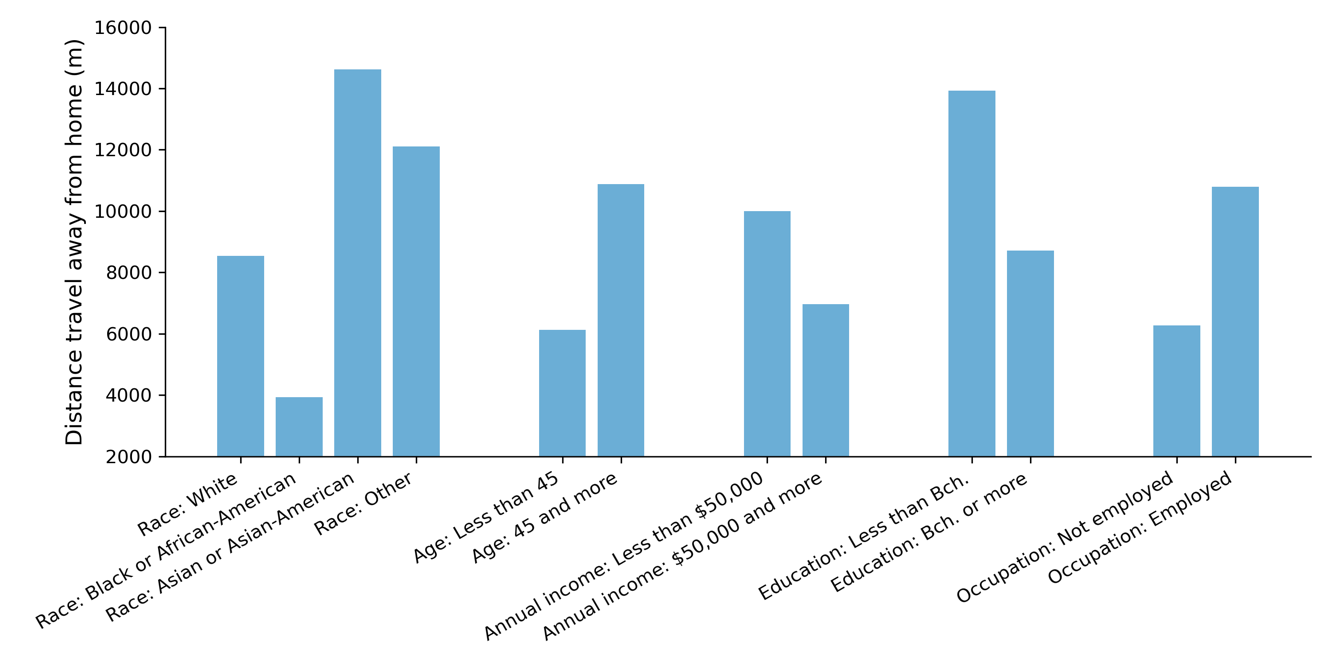


**Figure S9.** The average distance traveled from home (in meters) across various demographic categories (race, age, income, education, and employment). Each bar represents the mean distance for a specific subgroup.


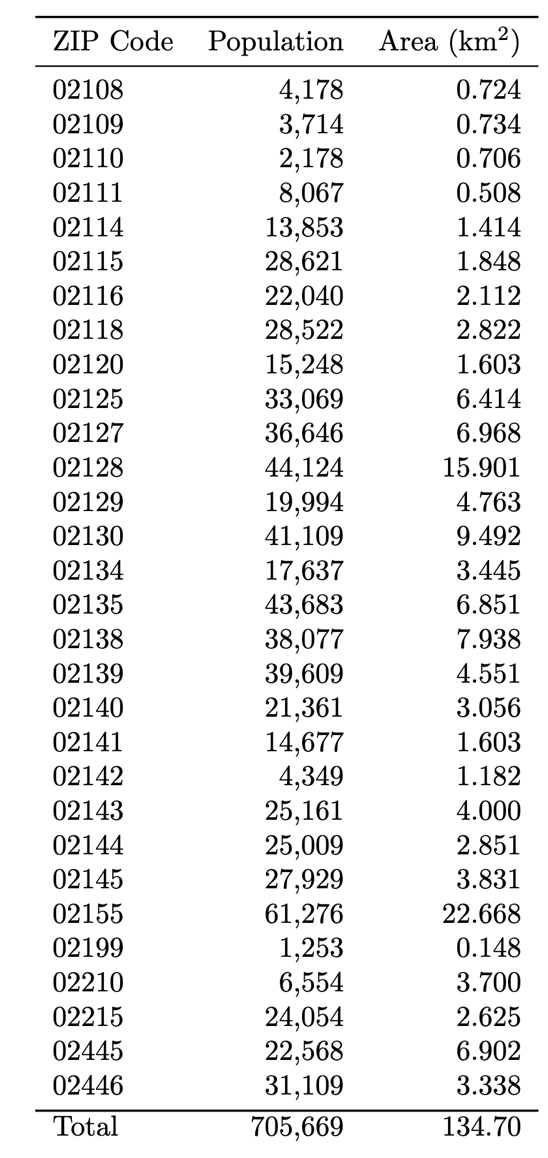


**Table S1.** Population and area for the 30 most visited ZIP codes in Boston Metropolitan Area


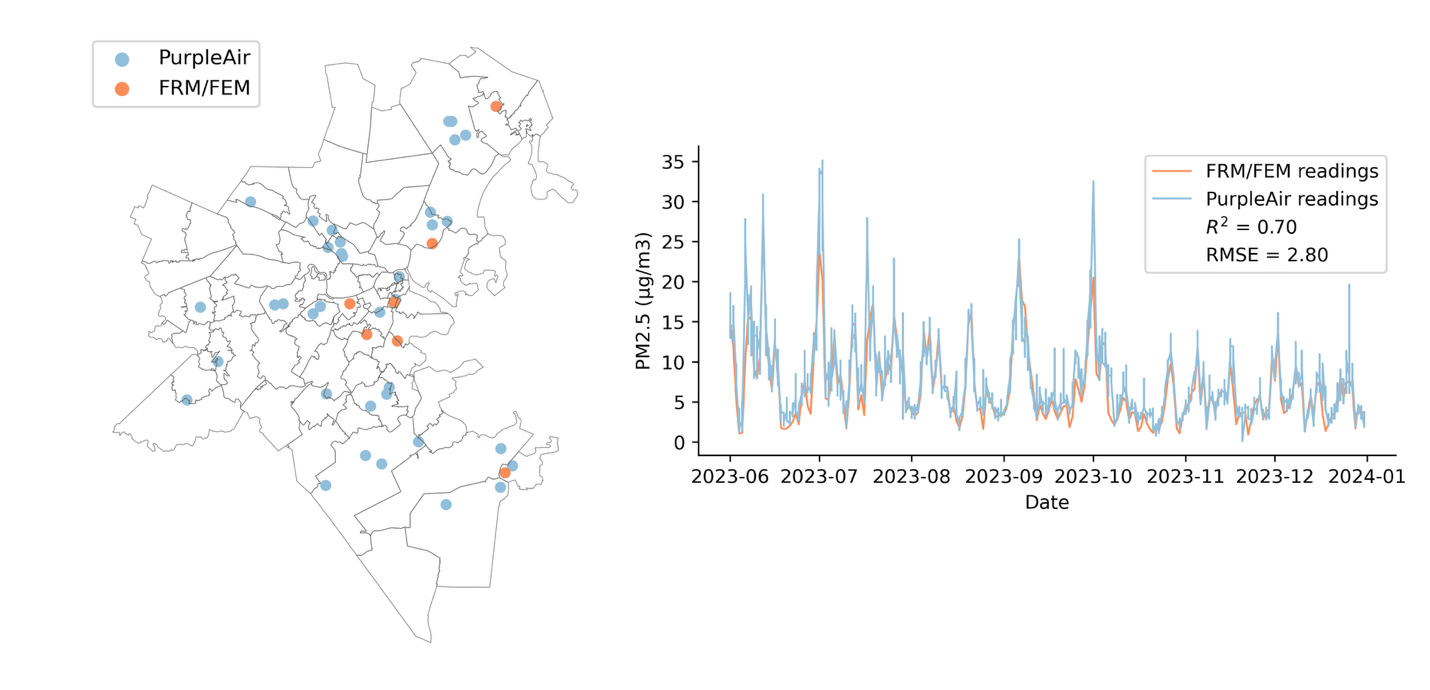


**Figure S10.** Comparison of daily PM_2.5_ averages between the PurpleAir monitors used in this study and the Federal Equivalent Method (FEM) and Federal Reference Method (FRM) monitors, which serve as the regulatory-grade reference. The low-cost PurpleAir monitors exhibit a similar daily trend, with a coefficient of determination R^2^=0.70 and a root mean square error (RMSE) of 2.80 µg/m^3^


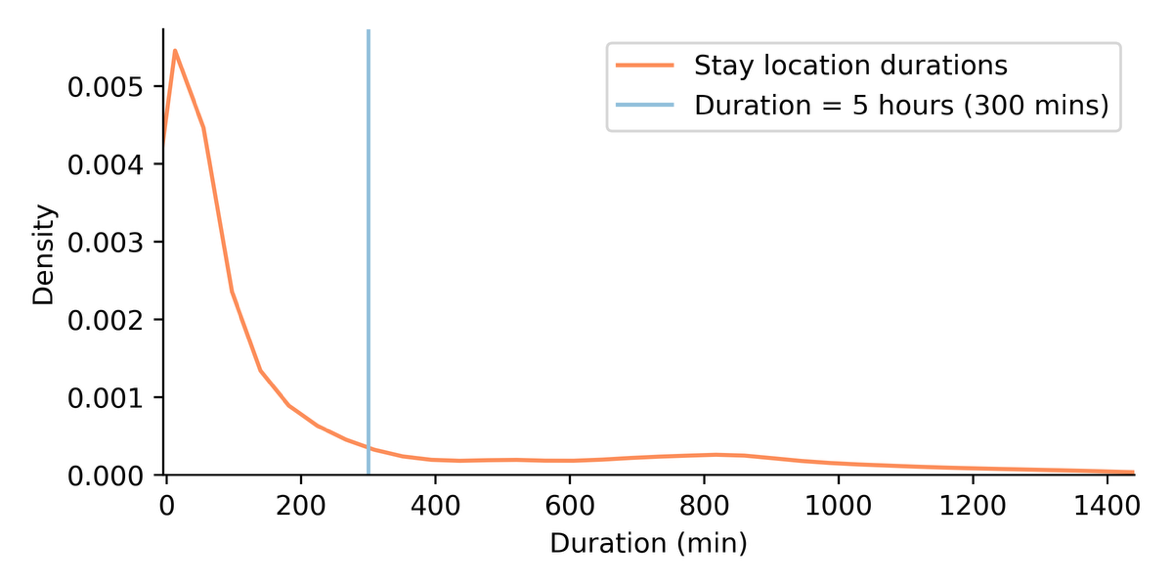


**Figure S11.** Distribution of stay durations in the BostonWalks dataset. To reduce the likelihood of including prolonged indoor stays, we applied a maximum duration cutoff of 5 hours. This exclusion criterion helps align our exposure estimates with outdoor PM_2.5_ concentrations, as measured by outdoor air quality monitors.
